# A State-of-the-Art Review on the Role of Cognitive and Motor Reserve on Quality of Life: A Focus on Cardiovascular Patients in a Lifespan Perspective

**DOI:** 10.3390/geriatrics9030059

**Published:** 2024-05-09

**Authors:** Jessica Giannì, Maura Crepaldi, Giulia Fusi, Francesca Colombi, Agostino Brugnera, Andrea Greco, Angelo Compare, Maria Luisa Rusconi

**Affiliations:** Department of Human and Social Sciences, University of Bergamo, 24129 Bergamo, Italy; jessica.gianni@unibg.it (J.G.); maura.crepaldi@unibg.it (M.C.); giulia.fusi@unibg.it (G.F.); francesca.colombi@unibg.it (F.C.); agostino.brugnera@unibg.it (A.B.); andrea.greco@unibg.it (A.G.); angelo.compare@unibg.it (A.C.)

**Keywords:** cognitive reserve, motor reserve, cardiovascular diseases, quality of life, aging

## Abstract

Cardiovascular diseases (CVDs) reflect a huge and diversified condition that influences patient quality of life (QoL) both in the physical and mental aspects, especially in older adults who often present comorbidities and may be affected by cognitive decline. The concept of cognitive reserve (CR), which is built through life course experiences, has widely been considered a protective factor against cognitive decline, while the results of QoL in the field of CVDs are still controversial. In particular, there is a lack of evidence that explicitly explores the effects of CR on the QoL in CVD cases since studies have considered only single CR proxies (e.g., education) or specific cardiovascular conditions. Moreover, none of them have considered the motor reserve (MR), another recent concept that considers the amount of physical activity carried out during a lifespan. Its potential role in preventing age-related diseases has been observed, but more clarification is needed given the importance of the physical component in CVDs. The present state-of-the-art review aims to (i) examine how the literature conceives CR and its proxies in CVDs relating to QoL and (ii) integrate the concept of MR in this framework. Implications for clinical practice will also be discussed.

## 1. Introduction

Cardiovascular diseases (CVDs) are a group of disorders of the heart and blood vessels that include conditions such as coronary heart disease, congenital heart disease or acute events like heart attacks and strokes. CVDs represent the leading cause of mortality worldwide, with an estimated 17.9 million deaths recorded in 2021 [1]. Patients affected by CVDs have implications both on their physical and emotional sphere; they experience physical symptoms such as fatigue, dyspnea or chest pain, which automatically impacts their emotional and social well-being, thus bringing a significant impairment in their quality of life (QoL) [2]. QoL is defined as “a broad-ranging concept affected in a complex way by the person’s physical health, psychological state, level of independence, social relationships and their relationship to salient features of their environment” by the World Health Organization [1]; another dimension closely linked to QoL but focused on health, which is widely considered in the field of CVDs, is health-related quality of life (HRQoL). This is defined as the individual’s functioning in life concerning their perceived well-being in physical, mental and social domains of health [3].

Currently, a great deal of effort is being made to understand how the burden of CVDs can be reduced; in fact, as the population ages, the global CVD burden is continuing to increase, especially among older adults due to their older age, multiple comorbidities and frailty [4]. It seems that a partial solution lies in including CVD management interventions at the primary care level, thereby making interventions more usable and accessible for everyone [1]. However, it represents only a partial solution as it acts when the pathological condition is already established; it would be useful to act, in a complementary way and at a preventive level, by an intervention on the involved risk factors (e.g., unhealthy diet, tobacco, harmful alcohol use and physical inactivity) [1]. Moreover, at an interventional level, the current strategies for the management of patients with CVDs are intended to reduce mortality and prolong survival, but treatments should be also focused on improving patient QoL [2]. On this note, cardiac rehabilitation programs focusing on lifestyle modification, psychological interventions and education seem to enhance patient QoL [5].

Starting from these premises, this review aims to highlight the possible role of two constructs: cognitive (CR) and motor reserve (MR). These correspond, respectively, to the individual number of cognitive and physical activities accumulated during the lifespan of CVD patients’ perceived QoL. We hypothesize that these two concepts would be fundamental to (i) operating preventively, thus constituting a protection and buffer effect to deal with the pathology, and (ii) influencing patient QoL when the pathology is full-blown, with a specific focus on the older population.

Insights into these two constructs will be discussed, and an overview of “if and how” CR and MR have been associated with QoL will be provided: first in the more general literature and then in CVD patients.

## 2. Cognitive Reserve Hypothesis

The concept of reserve was first developed following the observation of a non-linear relationship between brain pathology severity and its clinical manifestation, as well as a means to explain the inter-individual differences in how the brain responds to pathology. Thus, it is relevant when we refer to situations that imply the involvement of the brain in sustaining damage [6].

According to the cognitive reserve hypothesis conceptualized by Stern [6,7], CR is the “ability to optimize or maximize performance through differential recruitment of brain networks, which perhaps reflects the use of alternate cognitive strategies”. In other words, it describes the ability of the brain to use alternate paradigms to solve problems when the standard approach is no longer available [8].

The concept of reserve as it is meant in this article emerged from the development and combination of different models over time. Some of them, the so-called passive models of reserve and threshold models [9,10], conceived reserve as a passive process defined in terms of the amount of damage that can be sustained before reaching a critical threshold; from this point of view, the brain reserve capacity would depend on the number of neurons and synapses and on the brain size, which bring later clinical manifestations following brain damage. Otherwise, when following the active model [6,7], CR is not something predetermined but can be described as the accumulation of brain resources during an entire lifespan [11] that helps the brain to actively cope with or compensate for brain damage. Recently, researchers have assumed that passive and active models should be considered complementary because they provide complementary explanations of different forms of functional plasticity [6]. According to a more comprehensive point of view, there is another line of research that conceives reserve as a mechanism of pure defense expressed by the organism during evolution, which is what is meant when talking about “acquired resilience”. This study postulates that reserve has a simple tissue basis: it is the result of an evolved system of cellular mechanisms that allow mammals to use everyday stresses (e.g., exercise or caloric restriction) as stimuli to increase the resilience of tissues [12] (this would also include a brain-level reserve).

Delving into this aspect, to better understand the complexity of the specific CR hypothesis, three different concepts have been highlighted over time corresponding to the interrelated perspectives related to CR [11]: (i) Brain Reserve, which has already been defined; (ii) Brain Maintenance, which considers the development of age-related brain changes variability (physiological or pathological) due to genetics or lifestyle; and (iii) Compensation, which refers to the recruitment of neural resources in response to a certain cognitive demand. Moreover, according to this last facet, the literature also discusses the neural dimension related to CR, which refers to the inter-individual variability in the brain networks [13]. According to Stern and colleagues [14], changes in neural activity among individuals are, in fact, strongly related to different levels of CR, thereby helping to explain the individual differences in the capacity to cope with age-related changes or pathologic conditions.

### Measurement of Cognitive Reserve

Traditionally, attempts to operationalize CR have been made through the measurement of different proxies. In particular, variables that are descriptive of lifetime experience are commonly used as indicators of CR [7], where CR is considered a construct that is not directly observable and that it is quite complex to reach a quantitative measure of it.

One of the first indicators of CR was IQ or pre-morbid IQ [15,16]; this indicator considers the correlation between intelligence and CR, which is a correlation supported by the literature. Also, processing resources and executive functions have been taken into account as two of the most commonly used indicators of CR [16,17] together with performance on vocabulary tests and education [18]. A significant number of studies in the literature have focused on this last indicator, where it is considered that education is the number of years of completed study or the degree of literacy. Several studies conducted on normal aging and on patients affected by neurodegenerative diseases, such as Alzheimer’s disease (AD), have shown a more rapid cognitive decline in individuals with lower educational attainment [19,20], as well as greater levels of CR in AD patients with higher education, which acts as a protective factor against cognitive decline [15,21]. However, educational attainment as a proxy for CR presents several limitations as it might be particularly influenced by sociodemographic and cultural factors [22,23].

This holds true even for other demographic factors like occupational achievement. In this field, work complexity has been considered: different occupations require varied levels of mental demands, thereby providing heterogeneous forms of mental exercise that support brain functions [24,25,26]. Along with all these factors, it is logical to expect that other lifetime exposures could also be beneficial in increasing CR. Pre-morbid engagement in leisure activities seems to play a pivotal role in the construction of CR: intellectual, social and physical activities have been considered as part of leisure-time activities [7,27]. According to the last indicators defined, many studies have focused on education, occupation and leisure activities as the most frequently used proxies of CR [28].

Despite the attempts made thus far to operationalize and measure CR, it is important to consider that much of the literature agrees that CR is not a fixed factor [11]; therefore, it can continuously be modified by life experiences (i.e., through indicators previously mentioned or those that are a consequence of cognitive stimulation), even when the brain is already affected by neuropathology (i.e., through rehabilitation trainings [8,29,30]) and the main instruments adopted thus far for its measurement are not very sensitive to longitudinal changes.

## 3. Cognitive Reserve and Its Relationship with Quality of Life

Most of the CR literature concentrates on the effects on cognitive functioning, thereby showing what different levels of CR entail in the cognitive sphere. Thus, CR has been primarily investigated in dementia [8], acquired brain injury [31] and stroke [32]. The concept has also been extended to healthy aging and a broad range of neurological and psychiatric conditions. Its protective role in global cognitive functioning has been widely proven, both in healthy aging [6,11,33] and in a range of neurological conditions such as Alzheimer’s disease [34], Parkinson’s disease [35] or multiple sclerosis [36].

The relationship between CR and the psychological framework is less explored. Recent findings, in line with the previous literature, have shown a positive influence of CR on perceived psychological well-being in healthy older adults [37], thereby indicating that people with more years of work accumulated, who have held jobs requiring more cognitive and behavioral flexibility and are involved in diversified free time activities, perceive better coping strategies and have higher emotional competences. Another recent work by Porricelli and colleagues [38] explored the relationship between CR and mental health in healthy adults, and it showed that higher levels of CR corresponded to greater mental health when considering measures of anxiety, depression and stress. However, more knowledge in the field is required, especially regarding the effects of CR on QoL. From a speculative point of view, it is possible that people with greater levels of CR, who are supposed to be able to better cope with various disorders, could also experience a greater QoL. According to Lara and colleagues [39], who measured CR in a sample of older healthy adults by considering the three main indicators previously highlighted (education, work and leisure-time activities), a higher CR was associated with higher QoL. Furthermore, considering the health-related aspect of QoL in line with previous findings, a more recent work by Ihle and colleagues [40] considered the association between CR, cognitive functioning and health-related quality of life (HRQoL) levels on a sample of healthy older adults. They reported that better cognitive functioning together with higher CR (operationalized by education and cognitive level required at work) is fundamental to sustaining perceived good QoL, particularly for the mental component related to health status.

There are only a few studies that have focused on CR and QoL in clinical samples, and they have focused on heterogeneous pathologies such as psychiatric disorders [41], multiple sclerosis [42] and other medical conditions [43]. Contrasting results have emerged from these studies: Anaya and colleagues [41] found that a higher CR was positively associated only with the physical component of QoL and negatively with the mental component, thus indicating that psychiatric patients with bipolarism who have higher CR levels perceive better physical health, but they also show a worse subjective perception of their mental health than patients with lower CR. Conversely, Schwartz and colleagues [42] and Gomez-Beldarrain and colleagues [43], who analyzed patients with multiple sclerosis and with chronic migraine, respectively, found positive associations between CR and both components of QoL (i.e., physical and mental QoL). These contrasting results have arisen due to the heterogeneous samples analyzed and due to the use of different tools to measure QoL and CR. The majority of the studies on clinical samples and healthy elderly people usually measure QoL by dividing it into two sub-components, mental and physical QoL. One of the most used tools for measuring this in this field is SF-36 [44] (as well as its short version, SF-12). Others have used comprehensive tools such as WHOQOL-AGE [45], which is specific for the older adult population [39]. Again, other studies (e.g., [40]) have focused on a specific construct that is very close to QoL, which is HRQoL. This alternative metric reflects an individual’s functioning in life with respect to their perceived well-being in the physical, mental and social domains of health [3].

Another reason that brings contrasting results can be found in the use of different tools and methods to operationalize CR. Considering only the studies mentioned, we can see that none of them used the same tool and the same proxies; some used CR questionnaires [40], which considers a large number of proxies of CR together (i.e., level of education, parental level of education, training courses, occupation, musical training, languages, reading activities and intellectual games [46]), whereas others used more than one tool (i.e., CR questionnaires and CRI-q) to collect more indicators at a time [42,43]. Lastly, others have used only single proxies or the union of more indicators without a specific instrument (i.e., educational level and occupational attainment [40], as well as premorbid IQ [41]).

When taking these considerations together, it becomes especially difficult to compare studies where constructs are not homogeneous.

It is also necessary to consider that the relationship between CR and QoL is often mediated by other health-related factors such as depression, cognitive functioning, disability [39], health-related behaviors [42] and coping strategies [30]. Indeed, it has been shown that CR seems to influence those problematic health behaviors such as smoking and obesity, which may affect both the severity and the course of the disease and acts across a broad spectrum on patient QoL [42]. Furthermore, greater CR levels might allow individuals to use effective coping strategies to handle potentially stressful situations, such as, for example, those derived from their functional limitations [30]. Thus, it is useful to keep in mind that finding a direct relationship between these two constructs is challenging, especially when considering the number of factors that can mediate this relation and that these factors can vary depending on the clinical sample examined.

## 4. Cognitive Reserve and Quality of Life: A Focus on Patients with Cardiovascular Diseases

The problem relating to the operationalization of CR makes it difficult to analyze this construct, especially if we want to explore and relate it to huge and complex medical conditions like CVDs.

There is very little research in the literature available regarding CR on this cohort of patients; this is because most of the research focuses on alterations in the cognitive domain resulting from a cardiovascular event or a chronic condition and less on how a starting cognitive level or reserve can affect a patient’s well-being and QoL. Moreover, as we have seen in the last paragraph, QoL is investigated in different ways, or it is often replaced by HRQoL, especially in clinical samples. The last, but not least, point concerns the influence of those health-related factors that mediate the relationship between CR and QoL, thus making it challenging to reach homogeneous considerations.

Over the years, some attempts have been made to associate some proxies of CR with QoL in the field of CVDs. Most studies considered specific cardiac pathologies individually, while really few evidence considered the general cardiovascular framework.

Level of education as a predictor of QoL was the indicator mostly considered in the field of CVDs. For example, it has been analyzed considering samples of patients with heart failure (HF) [47,48]. Results showed that low education levels were associated with a worse QoL; moreover, from a longitudinal point of view, they found better QoL for highly educated patients in both physical and functional domains.

From a wider point of view, other findings have confirmed the role of higher educational levels in cardiac patient QoL, and occupational status has also been considered [49,50]. However, the findings about this last proxy are ambiguous; other empirical evidence, indeed, has showed no relationship between one’s occupation and QoL, such as, for example, in patients with myocardial infarction (MI) [51]. It means that further clarification on the role of occupational status in CVDs is still needed.

Moreover, it is necessary to increase studies in the field to clarify the predictors of QoL in this cohort of patients. A proposal aimed in this direction could try to quantify CR with respect to QoL by considering homogeneous samples and methodologies. It would be a turning point for knowledge in the field, representing the starting point of planning clinical interventions based on CR, which is modifiable during a lifespan and, hypothetically, directly acts to promote patient QoL.

However, according to this aim, we ought to consider factors that influence the relationship between CR and QoL in cardiovascular patients, particularly the role of cognitive functioning and health-related behaviors. In fact, from a physiological point of view, modified cardiovascular conditions can induce changes in cerebral perfusion, which is one of the determinants of cognitive deterioration [52]. Attention, working memory, executive function and psychomotor speed seem to be mostly affected by cardiovascular events. Moreover, a growing line of evidence shows the importance of the cardiovascular system for the pathogenesis of dementia [53,54,55]. According to this evidence, the cognitive decline that arises from some form of dementia is caused by a clinically silent bleeding of small cerebral vessels; such a hemorrhage seems to be induced by a long exposure to the stress of the pulse. With age, in fact, the arterial tree stiffens, and the intensity of the pulse grows. Therefore, it emerges that the pulse becomes more intense and destructive with age, thus causing vascular breakdown, a progressive loss of neurons and, consequently, cognitive decline [54].

The alteration of the cognitive domain represents, in turn, an important factor that can limit a patient’s ability to follow correct health-related behaviors (i.e., following their dietary or medication regimes), thus further reducing their QoL [56]. Following this line, in a recent review by Zaben and Khalil [57] that was conducted on patients with acute coronary syndrome (ACS), the importance of self-behavior was highlighted together with health literacy for patient QoL. Therefore, according to this evidence, good general executive functioning is crucial for the relationship between cognitive domain and QoL, as well as the motor reserve (MR), which will be shortly discussed. Furthermore, from a lifespan perspective, executive functioning is often the first domain to be impaired with aging, thereby contributing to cognitive impairment, loss of autonomy and reduction in QoL [58,59].

## 5. Motor Reserve: Construct Definition

The MR is a relatively recent concept in the literature, and it refers to the flexible and dynamic construct that potentially increases over time and compensates for age-related motor and cognitive loss [60]. It is another kind of reserve, and it is built through the accumulation of physical activity carried out throughout life; it has emerged from studies on clinical samples, particularly with respect to patients affected by neurodegenerative disorders such as Parkinson’s disease (PD)—a pathological neurologic condition that brings important implications on patient motor functioning. It seems that a higher MR is associated with a greater ability to cope with pathological motor skill decline, both in terms of it as a pathological condition and in healthy late adulthood [61]. To better clarify what we intend with MR, it is necessary to highlight that physical activity’s beneficial effects derive from it being carried out regularly over time [60]; without consistency and frequency, it would not be possible to build a strong reserve, and it would correspond only to a sporadic physical exercise, thus leading to insufficient physiological changes [62].

Starting from studies about patients with PD, it has emerged that some proxies contribute to the enhancement of MR, including dominant side-laterality [63], educational attainment [64] and premorbid exercise engagement [65]; such proxies seem to allow patients to better cope with PD-related pathological deficits. Over the years, there is more evidence of attempts to gradually separate the concept of MR from PD and to understand its functioning from a more general point of view. In a recent work by Pucci and colleagues [60], the potential effect of MR on the cognitive functioning of a sample of healthy individuals over 50 years was explored, where the researchers tried to operationalize MR in a specific way. They considered the amount of physical activity (PA) and physical exercise (PE) carried out by individuals, and they defined PA as the result of unstructured daily activities in various contexts such as work, housekeeping, walking and leisure [11,66], and they defined PE as all movements produced by skeletal muscles resulting in energy expenditure, including structural physical activities [67]. According to this evidence, an active lifestyle and engagement in structured PA help to keep the body healthy, but it also helps to guarantee an effect on the brain, cognition and mood [60].

In line with that, as well as considering that a large part of the population is currently low-active or sedentary and are engaged in levels of physical activity that are insufficient for health gain [68], there are guidelines that support the importance for older adults to engage in exercise training to promote both physical and cognitive health [69]. Recommendations suggest that a program of regular exercise including cardiorespiratory, resistance, flexibility and neuromotor training can improve physical fitness and is essential for health status vitality in most adults [70]. Moreover, according to the World Health Organization [71], it seems that adults aged 65 and older should perform at least 150 min per week of moderate-intensity activity or 75 min of vigorous-intensity activity. Thus, regular physical activity of moderate intensity has been recognized as a significant beneficial factor for health, both reducing the risk of developing CVDs (i.e., heart disease, stroke, hypertension, type 2 diabetes, etc.), psychiatric symptoms such as depression and anxiety [72,73], and also sustaining older adult cognitive functioning [69].

The relationship between physical exercise and cognition is complex, physiological changes have been proposed as preceding changes in cognition [74], and this is why a longer intervention is needed to observe a significant change in global cognition.

Physical activity is important because it seems to slow down the process of age-related neuronal and volumetric loss, and it reduces both lesions in white matter and myelin loss, thus promoting better oxygenation and blood supply to the brain [75]. Moreover, it maintains the neural network through neuroplasticity, brain perfusion and neurogenesis; as such, people with good physical fitness can tolerate a greater neuropathological burden without suffering cognitive impairment [76]. Recent evidence has highlighted its beneficial effects, especially on some cognitive abilities (i.e., executive functions, learning, memory and language) in the older population [77]. Executive functions, which are supported by lifestyles including physical activity, play a key role in the elderly’s mental health, as higher levels of cognitive processes are fundamental in targeting goals, effortful behavior and environment adaptation [78,79]. In fact, currently, physical activity programs are well-established strategies for improving working memory, cognitive flexibility and inhibitory control in cognitively healthy older adults [80].

If we try to compare the two types of reserve analyzed, it is interesting to assume that the factors that contribute to increasing MR might also increase CR; however, factors that determine a high CR (i.e., education, work and cognitively stimulating leisure activities) do not necessarily lead to an increase in MR [60]. This leads to the conclusion that they are two different and independent types of reserve, both contributing to good cognitive functioning differently.

## 6. Motor Reserve and Quality of Life

Engaging in regular physical activity, beyond the delay of age-related physiological and cognitive decline and a reduction in the risk of developing common diseases, seems to also improve subjective QoL [73,81,82]. These findings are supported by other evidence, thus indicating that MR promotes better general cognitive functioning and QoL [83]. Any type of physical activity implies, in fact, a connection between individuals and their own body with the environment involving numerous and coordinated actions, all of them requiring cognitive functioning (i.e., the perception and cognitive estimation of distance, size, shape and weight of objects and spaces, which modulates the motor response and calibrates the action within the surrounding space) [60]. Therefore, maintaining an active lifestyle in older age in terms of cognitively stimulating activities and regular physical activity across a lifespan is advantageous from multiple points of view. According to the World Health Organization, physical activity is recognized as relevant for supporting “healthy aging” in different ways, particularly as a key enabler of social participation, personal autonomy and greater psychological well-being and QoL [84].

Along with QoL, research has shown that engagement in structured physical activity protocols can improve some aspects of psychological well-being in the elderly, such as mood [85,86], self-perception [87], depression [88], emotional well-being and decreased anxiety [70]. According to these findings, the literature remains sparse, with most studies conducted to investigate the impact of structured programs of exercise on mental well-being. A growing number of studies, in fact, have focused on exercise training even in cohorts of patients with hemodialysis [89,90] and with multiple sclerosis [91], thereby showing that regular physical activity improves mental and physical QoL. In contrast, very little is known about the influence of daily physical activity accumulated over time. That is why it is necessary to consider the nascent concept of MR in the field of mental well-being and QoL, especially in clinical samples that experience an impairment of QoL, like patients affected by CVDs.

## 7. Conclusions

According to the literature reviewed in this study, CR and MR are two predictors of better cognitive functioning. Preserving good cognitive functioning is crucial to support daily activities and to maintain autonomy and QoL, especially in older adults who often present multiple comorbidities. Studies that have tried to link CR and MR with QoL in healthy adults and heterogeneous clinical samples have demonstrated a great deal of difficulties and methodological limitations, which have brought contrasting results.

The field of CVDs, a group of disorders representing the leading cause of mortality worldwide, is even more involved due to the variety of physical symptoms that influence patient QoL. There are few and inconsistent studies about the link between CR, MR and QoL in this cohort of patients: some studies have individually considered one or two CR proxies (i.e., education or occupation), or they have considered only a single pathologic condition that is included in CVDs. Regarding MR, most of the evidence is focused on the effects that physical activity programs have on patient QoL; thus, the influence of daily physical activity accumulating over time from a preventative point of view has been neglected. According to such considerations, it has emerged that there is further evidence focused on relating CR and MR in CVDs. As defined in the literature reviewed, the QoL of CVD patients can be useful for implementing knowledge in the field and to bring benefits in clinical practice, mainly in terms of primary and secondary, but also tertiary, prevention interventions. Indeed, considering that CR and MR can always be expanded and potentiated, preventive interventions to enhance the two types of reserve can be extremely useful in supporting CVD patients’ cognitive and psychological resources. They could be useful for providing patients with more resources to act preventively on the risk factors of CVDs and for overcoming their occurrence, but also by acting retrospectively to improve their chances of responding positively and flexibly to the difficulties encountered during the disease. These resources might, therefore, potentially have significant beneficial effects on patient perception of QoL, thus also contributing to other health-related dimensions such as the ability to regulate their emotions or adherence to therapies. Future studies may clarify the relationship between these variables.

## Data Availability

Any dataset was used or created in this state-of-the-art review so no data is available.
